# Putative COVID-19 therapies imatinib, lopinavir, ritonavir, and ivermectin cause hair cell damage: A targeted screen in the zebrafish lateral line

**DOI:** 10.3389/fncel.2022.941031

**Published:** 2022-08-24

**Authors:** Allison B. Coffin, Emily Dale, Emilee Doppenberg, Forrest Fearington, Tamasen Hayward, Jordan Hill, Olivia Molano

**Affiliations:** ^1^Department of Integrative Physiology and Neuroscience, Washington State University, Vancouver, WA, United States; ^2^College of Arts and Sciences, Washington State University, Vancouver, WA, United States

**Keywords:** COVID-19 therapy, hair cell, zebrafish, lateral line, ototoxicity, ivermectin, remdesivir

## Abstract

The biomedical community is rapidly developing COVID-19 drugs to bring much-need therapies to market, with over 900 drugs and drug combinations currently in clinical trials. While this pace of drug development is necessary, the risk of producing therapies with significant side-effects is also increased. One likely side-effect of some COVID-19 drugs is hearing loss, yet hearing is not assessed during preclinical development or clinical trials. We used the zebrafish lateral line, an established model for drug-induced sensory hair cell damage, to assess the ototoxic potential of seven drugs in clinical trials for treatment of COVID-19. We found that ivermectin, lopinavir, imatinib, and ritonavir were significantly toxic to lateral line hair cells. By contrast, the approved COVID-19 therapies dexamethasone and remdesivir did not cause damage. We also did not observe damage from the antibiotic azithromycin. Neither lopinavir nor ritonavir altered the number of pre-synaptic ribbons per surviving hair cell, while there was an increase in ribbons following imatinib or ivermectin exposure. Damage from lopinavir, imatinib, and ivermectin was specific to hair cells, with no overall cytotoxicity noted following TUNEL labeling. Ritonavir may be generally cytotoxic, as determined by an increase in the number of TUNEL-positive non-hair cells following ritonavir exposure. Pharmacological inhibition of the mechanotransduction (MET) channel attenuated damage caused by lopinavir and ritonavir but did not alter imatinib or ivermectin toxicity. These results suggest that lopinavir and ritonavir may enter hair cells through the MET channel, similar to known ototoxins such as aminoglycoside antibiotics. Finally, we asked if ivermectin was ototoxic to rats *in vivo*. While ivermectin is not recommended by the FDA for treating COVID-19, many people have chosen to take ivermectin without a doctor’s guidance, often with serious side-effects. Rats received daily subcutaneous injections for 10 days with a clinically relevant ivermectin dose (0.2 mg/kg). In contrast to our zebrafish assays, ivermectin did not cause ototoxicity in rats. Our research suggests that some drugs in clinical trials for COVID-19 may be ototoxic. This work can help identify drugs with the fewest side-effects and determine which therapies warrant audiometric monitoring.

## Introduction

Effective therapies for COVID-19 are critical to reduce the disease burden from this global pandemic. Some studies repurpose existing FDA-approved drugs such as viral protease inhibitors, particularly HIV drugs such as lopinavir and ritonavir, broad-spectrum antibiotics such as azithromycin, or antivirals such as favipiravir and remdesivir (Asai et al., 2020; Gordon et al., 2020; Guy et al., 2020; Yadav et al., 2020). Conversely, other trials test new drugs based on SARS-CoV-2-specific targets (Asai et al., 2020; Favalli et al., 2020; Tarasova et al., 2020). While many trials target viral entry or replication pathways, other trials seek to reduce the over-active immune response seen in some COVID-19 patients; a positive feedback loop known as a cytokine storm (Coperchini et al., 2020; Horby et al., 2020; Mehta et al., 2020; Quirch et al., 2020). Some immune-focused trials use drugs that specifically target interleukins or other cytokines while other trials take a different approach using non-specific anti-inflammatories. There are over 900 drugs in clinical trials for COVID-19 as of April 2022 (Clinicaltrials.gov, 2022). While this pace of drug development is necessary, it also comes with an increased risk of producing therapies with significant side-effects, including hearing loss.

Several drugs in clinical trials for COVID-19 are implicated in ototoxicity (Awad and Naimark, 2020; De Luca et al., 2021). For example, the macrolide antibiotic azithromycin is linked to hearing impairment, although this link is based primarily on case reports and some studies are contradictory [Lo et al., 1999; Alrwisan et al., 2018; Pomares et al., 2018; reviewed in Coffin et al. (2021)]. Other case reports suggest that hearing loss is correlated with the use of protease inhibitors [Williams, 2001; reviewed in Coffin et al. (2021)]. Many COVID-19 drugs are given to patients with substantial immune activity, including cytokine storms, and immune activation potentiates auditory toxicity of known ototoxins such as cisplatin and aminoglycosides (Oh et al., 2011; Hirose et al., 2014; Koo et al., 2015). This evidence suggests that immune activation will increase drug ototoxicity in some patients. Despite the risk for ototoxicity, very few studies examine hearing in COVID-19 patients.

Audiometric monitoring in clinical trials is expensive, difficult to coordinate across hospitals, and complicated by factors specific to different patient populations. For example, many patients who contract severe COVID-19 disease are elderly and likely have a degree of pre-existing age-related hearing loss, making it more difficult to detect moderate drug-induced hearing loss. Furthermore, while rare, some case reports suggest that SARS-CoV-2 infection can cause hearing loss, obscuring the distinction between hearing loss due to disease and hearing loss due to treatment (Koumpa et al., 2020; Maharaj et al., 2020). Pre-clinical studies in animal models are therefore needed to determine the ototoxic potential of COVID-19 therapies in order to identify drugs that warrant audiometric monitoring in patient populations. Here, we use the zebrafish (*Danio rerio*) lateral line as a model for hair cell toxicity.

The lateral line comprises a series of externally located sensory organs (neuromasts) on the head and body of the animal that each contain sensory hair cells interdigitated with supporting cells. Fish use their lateral line system to detect nearfield water movement for behaviors such as prey detection, predator avoidance, orientation to current, and schooling (Coombs et al., 2014). Critically, lateral line hair cells are homologous to mammalian inner ear hair cells and at 5 days post-fertilization (dpf), zebrafish hair cells show mammalian-like responses to known ototoxins (Harris et al., 2003; Coffin et al., 2004; Santos et al., 2006; Ou et al., 2007). The zebrafish lateral line is ideal for pharmacological manipulation and *in vivo* visualization when compared to more traditional inner ear models, combining the accessibility of an *in vitro* preparation with the intact physiology of an *in vivo* system. Our group and others have previously used this model to screen drug and chemical libraries for hair cell toxicity (Chiu et al., 2008; Hirose et al., 2011; Neveux et al., 2017). Recently, Davis et al. (2020) demonstrated that chloroquine and hydroxychloroquine, two anti-malarials previously considered for COVID-19, were toxic to lateral line hair cells. Chloroquine was also ototoxic in neonatal mouse cochlear explants (Davis et al., 2020), further demonstrating that findings in zebrafish translate to mammalian systems.

We selected seven drugs in clinical trials for COVID-19 patients: lopinavir, ritonavir, azithryomycin, imatinib, remdesivir, dexamethasone, and ivermectin. We focused on pharmaceuticals in multiple COVID-19 trials with potential ototoxicity based on case reports and/or prior pre-clinical studies. Lopinavir and ritonavir are viral protease inhibitors used to treat HIV and are generally administered concurrently. A previous case report suggests the potential for lopinvir-ritonavir ototoxicity in HIV patients and ritonavir is toxic to auditory cells *in vitro* (Williams, 2001; Thein et al., 2014). The macrolide antibiotic azithromycin is associated with temporary hearing impairment in some HIV patients or those with certain lung conditions (Tseng et al., 1997; Albert et al., 2011; Rybak et al., 2021). Multiple case reports describe sensorineural hearing loss in patients receiving imatinib, a tyrosine kinase inhibitor with anti-inflammatory properties (Attili et al., 2008; Lin et al., 2012; Wasif et al., 2016).

Both remdesivir and dexamethasone received emergency use authorization in the United States for hospitalized COVID patients with severe disease. Remdesivir is an antiviral adenosine nucleotide analog and related analogs (e.g., ribavirin) are associated with hearing loss (Jain et al., 2011). On the other hand, the anti-inflammatory agent dexamethasone is widely used to treat sudden sensorineural hearing loss and several studies show that dexamethasone is not ototoxic (Egli Gallo et al., 2013; Hayward et al., 2019; Singh and Kumar Irugu, 2020; Hu et al., 2021). We therefore included dexamethasone as a negative control. Finally, we examined ivermectin, an anti-parasitic agent used in several early clinical trials for COVID. Little credible evidence exists for ivermectin as a beneficial COVID-19 therapy and multiple studies have been retracted for both scientific and ethical concerns, leading the FDA to warn patients against off-label use of ivermectin (López-Medina et al., 2021; Vallejos et al., 2020; Dyer, 2022; Meyerowitz-Katz et al., 2022). However, ivermectin has been promoted on “alternative” websites and gained notoriety on social media as a COVID-19 treatment. Based on this misinformation, patients are seeking ivermectin from often dubious sources, emphasizing the need to determine the ototoxic potential of this drug.

We found that lopinavir, ritonavir, ivermectin, and imatinib were all toxic to zebrafish hair cells. Our data suggest that several COVID-19 therapeutics have the potential to cause hearing impairment and that audiometric monitoring is warranted in patients receiving these potentially ototoxic medications.

## Materials and methods

### Zebrafish hair cell toxicity experiments

We used wildtype zebrafish of the *AB strain for the bulk of the experiments. *Tg(myo6b:ribeye a-GFP*) (a kind gift from K. Kindt at the NIDCD, hereafter called Rib-GFP) transgenic fish were used for experiments that visualized ribbon synapses, as described in the text. All fish were obtained from paired or group matings in the Coffin Lab zebrafish facility at Washington State University Vancouver. Larvae were reared at 28°C in standard fish water (pH ∼7.3, conductivity 900–1,100 μSiemens) with a 14:10 h light:dark cycle (Westerfield, 2007). At this age it is not possible to determine fish sex, but based on our experience, we expect approximately equal sex ratios. All experiments were approved by the Institutional Animal Care and Use Committee at Washington State University (Protocol 6024).

#### Drug treatments and hair cell assessment

We used 5–6 dpf zebrafish for all experiments. Experiments were conducted in E2 Embryo Medium (EM; 1 mM MgSO4, 120 μM KH2PO4, 74 μM Na2HPO4, 1 mM CaCl2, 500 μM KCl, 15 mM NaCl2 in distilled water at a pH of 7.2; Westerfield, 2007).

Hair cell damage was first assessed in wildtype *AB larvae. Prior to drug treatment, fish were incubated in DAPI (catalog # MBD0015, Sigma-Aldrich, St. Louis, MO, United States; diluted to 1 μg/ml in EM) for 10 min at 28°C. In live larvae, DAPI specifically labels lateral line hair cells and the label is retained after euthanasia (Uribe et al., 2018; Coffin et al., 2021; Holmgren et al., 2021; Lee et al., 2022; also see Supplementary Figure 1). Fish were then rinsed twice in EM and incubated for 24 h with variable concentrations (0.01–50 μM) in one of the following drugs (obtained from Sigma- Aldrich unless specified): remdesivir (catalog # 7226, Bio-Techne, Minneapolis, MN, United States), lopinavir, ritonavir, imatinib (catalog # S1380, S1185, S2475, respectively; Selleckchem, Houston, TX, United States), dexamethasone, azithromycin, or ivermectin (catalog # D1756, PHR1088, I8898, respectively). Control fish received equivalent volumes of vehicle (methanol for dexamethasone, DMSO for all other drugs). All drug stock solutions were diluted to the desired concentration in EM. Fish were then euthanized in 0.002% buffered MS-222 (Syndel, Ferndale, WA, United States) and fixed in 4% paraformaldehyde (PFA) (ThermoFisher, Waltham, MA, United States) for 2 h at room temperature or overnight at 4°C. Fish were then rinsed 2× in phosphate-buffered saline (PBS) and stored at 4°C in PBS:glycerol (1:1). Fish were mounted on double bridged coverslips in a drop of PBS:glycerol. Total hair cells in neuromasts IO1, IO2, IO3, OP1, and M2 (Raible and Kruse, 2000) were counted on a Leica DMRB compound microscope using a 40× objective. We selected these five head neuromasts because they are viewable in the same field of view and we have assessed these neuromasts in our previous ototoxicity research, allowing for comparisons across studies (Coffin et al., 2013; Uribe et al., 2018). Prior research on known ototoxins does not show differences in damage responses across neuromasts (Harris et al., 2003). As these neuromasts contain different numbers of hair cells, we summed the values from the five neuromasts to arrive at one value per animal.

#### Synaptic ribbon assessment

In Rib-GFP transgenic fish, the pre-synaptic ribbon protein ribeye is tagged with green fluorescent protein (GFP). We therefore used this fish line to assess ribbon damage following COVID-19 drug treatment. Fish were live-labeled with DAPI and treated with COVID-19 drugs as described above. After euthanasia and fixation in 4% PFA, we amplified the GFP signal with anti-GFP. Fish were rinsed in PBS, blocked in PBS with 0.1% Triton-X (PBST) and 10% normal goat serum, then incubated at 4°C overnight with anti-GFP (1:250 dilution, catalog # A-11122, Invitrogen, Waltham, MA, United States). Fish were rinsed in PBST, then incubated in goat anti-rabbit 488 secondary antibody (catalog # A11008, Invitrogen), followed by additional PBST and PBS rinses. We used a Leica SP8 confocal microscope to image the IO2, IO3, and M2 neuromasts, using a 63× oil objective and 3× digital zoom, then generated a maximum-point projection of each neuromast. We counted the total number of hair cells (DAPI + nuclei) and number of synaptic ribbons (GFP + punctae), then divided the number of ribbons by the number of hair cells to calculate the number of ribbons per hair cell.

#### Cell death assay

We used a TUNEL assay to quantify apoptotic cells in drug-treated fish (ApopTag Red kit, catalog # S7165, EMD Millipore, Burlington, MA, United States). Fish were live-labeled with DAPI, incubated in COVID-19 drug for 24 h and euthanized as described above. The fish were then fixed in 4% PFA for 2–3 h at room temperature, then rinsed twice in PBS and either stored at 4°C in PBS before TUNEL labeling (for fish labeled within 2–3 days of euthanasia) or stored in 1:1 PBS:glycerol (for fish stored for 4 days or more before labeling). For fish stored in PBS: glycerol, we first rinsed them twice in PBS, post-fixed for 10 min in 4% PFA, then rinsed twice more in PBS before proceeding with the TUNEL labeling protocol. The post-fixation step was used to preserve the DAPI labeling following glycerol storage.

Fish were labeled using slight modifications of our published protocol (Uribe et al., 2018). All steps were performed at room temperature unless specifically noted. Fish were incubated in 20 μg/ml proteinase K (Sigma-Aldrich) for 10 min at 37°C, rinsed in PBS for 5 min, treated with glacial acetic acid in ethanol (1:2 by volume) for 5 min, and rinsed twice in PBS. Next, fish were incubated for 30 s with 75 μl of equilibrium buffer, then incubated at 37°C for 60 min in working strength TdT enzyme (77 μl reaction buffer with 33 μl TdT enzyme). The reaction was stopped with stop/wash buffer (10 min), followed by three PBS rinses. Fish were then incubated in blocking solution with anti-digoxigenin (68 and 62 μl, respectively) for 1 h at 37°C, rinsed four times in PBS, and stored in PBS:glycerol prior to imaging. We imaged three head neuromasts per fish on a Leica SP8 confocal microscope using the 20× objective and 5× digital zoom. We generated a maximum-point projection of each neuromast and quantified the number of TUNEL+ hair cells (DAPI+/TUNEL+) and the number of TUNEL+ non-hair cells (DAPI-/TUNEL+) per image. The total region of interest was 62 μm × 62 μm per image, with each image containing a single neuromast and surrounding non-neuromast tissue. We then summed these values for each animal.

#### Mechanotransduction inhibition assay

Ototoxic aminoglycoside antibiotics primarily enter hair cells through the mechanotransduction (MET) channel located at the tips of the stereocilia (Marcotti et al., 2005; Alharazneh et al., 2011). We therefore inhibited MET channel activity using amiloride (catalog # A7410, Sigma-Aldrich), a known MET channel blocker that protects hair cells from aminoglycoside toxicity (Jørgensen and Ohmori, 1988; Coffin et al., 2009). Our preliminary experiments showed that amiloride treatment over 24 h was somewhat toxic to hair cells and that 1 h of treatment with COVID-19 drug (lopinavir or ritonavir) was not sufficient to cause hair cell death (data not shown). Therefore, we assessed hair cell death after 6 h of COVID drug exposure to identify a suitable time course for the subsequent amiloride experiments. Six dpf zebrafish were live-labeled with DAPI, then incubated in 0.1–200 μM of COVID-19 drug. Hair cells were quantified as described above.

We selected a concentration of each COVID-19 drug that yielded significant hair cell damage following a 6-h incubation, with no detectable organismal toxicity. Six dpf zebrafish were live-labeled with DAPI, incubated in 1 mM amiloride for 15 min, and then incubated in 1 mM amiloride and COVID drug for 6 h. We used the aminoglycoside 100 μM gentamicin as a positive control (Coffin et al., 2009). Hair cells were quantified as described above.

### Rat ototoxicity experiments

We examined the ototoxic potential of ivermectin in a rat *in vivo* model. Four-week-old Fischer 344 (CDF) rats of both sexes were obtained from Charles River Laboratories (Wilmington, MA, United States) and held in the rodent vivarium at Washington State University Vancouver for 2-3 weeks prior to use. Rats were housed two per cage with animals of the same sex with unlimited access to chow (Mazuri rat and mouse diet, St. Louis, MO, United States), water, and environmental enrichment. They also received one Fruit Loop per day. All experiments were approved by the Institutional Animal Care and Use Committee at Washington State University (protocol 6603).

#### Auditory physiology

We recorded auditory brainstem responses (ABRs) to quantify hearing thresholds. Rats were anesthetized with an intraperitoneal (i.p.) injection of 35 mg/kg ketamine and 3.5 mg/kg xylazine (females) or 60 mg/kg ketamine and 6 mg/kg xylazine (males) (ketamine and xylazine from Patterson Veterinary Supply, Devens, MA, United States). Rats were then placed in a sound attenuation chamber and subdermal needle electrodes were placed on the right mastoid, left mastoid, and vertex (recording, reference, and ground electrodes, respectively). The active electrode was connected to the positive input of a Dagan 2400 differential amplifier.

Tones were presented with a calibrated leaf-tweeter speaker (LCY K100; Ying Tai) placed 10 cm away from the right ear and 45° away from the midline. The speaker was calibrated over a range of 3–100 kHz before each recording session using a microphone (model 4135, Brűel & Kjaer) positioned 10 cm from the speaker where the animal’s right ear would be located. Acoustic output was delivered by a 16-bit digital to analog converter (500,000 samples/s, National Instruments) routed to a programmable attenuator (PA-5, Tucker Davis Technologies), then further routed to the speaker. Acoustic stimulation and data acquisition were controlled *via* custom-created software (Felix et al., 2019).

We used pure tone stimuli (18, 16, 24, 32 kHz, 5 ms duration, 1 ms rise/fall time, 10 rep/s), with alternating polarity to reduce stimulus artifact/cochlear microphonics in the final average waveform (Felix et al., 2019). Initial tones were played at 60 dB, with intensity adjusted by 10 dB steps. As the waveform diminished we decreased to 3 dB steps and averaged the response of 500 stimulus presentations per frequency and intensity level. Thresholds were visually determined based on the waveform, which was interpreted by two researchers blind to treatment. Threshold was defined as the lowest dB at each frequency with some distinguished peaks (Felix et al., 2019). Following ABR measurements, rats were administered the anesthetic reversal agent Antisedan (5 μg/rat, i.p. injection; Patterson Veterinary Supply). Rats were allowed to recover on a heating pad until they resumed normal activity, at which point they were returned to their home cage. ABR responses were recorded at baseline, then again following ivermectin treatment.

#### Ivermectin treatment

Ivermectin treatment was initiated approximately 1 week after ABR recording. Ivermectin stock was prepared in DMSO, then diluted in sterile saline. Rats were subcutaneously injected with 0.2 mg/kg ivermectin or an equivalent volume of saline with DMSO (vehicle control). The ivermectin dose was selected based on clinical trials registered by the summer of 2021 (e.g., NCT04391127, NCT04646109, NCT04591600). Clinical trials varied in the length of ivermectin administration; treatment studies in COVID-19 patients administered ivermectin for 5–7 days [reviewed in Cobos-Campos et al. (2021) and Cruciani et al. (2021)], while some prophylactic trials used a 2-week time course (Babalola et al., 2021). Further, self-administration of ivermectin was common in 2021 and there is little data on the time course used by individuals not under physician care. We therefore selected a 10-day time course of ivermectin; within the range of ivermectin clinical trials and consistent with studies using known ototoxins (Chowdhury et al., 2018; Kitcher et al., 2019).

Ivermectin injections occurred in the afternoon, during the rats’ active period, daily for 10 days, alternating injection sites between the left and right sides. Rats were weighed every other day to calculate appropriate drug dose and track weight gain. Following ivermectin treatment, rats were allowed to recover for 2 weeks prior to ABR recordings. Research using known ototoxins such as aminoglycosides demonstrates that larger (temporary) threshold shifts occur immediately following ototoxin exposure, while permanent threshold shifts stabilize within 2 weeks after cessation of drug administration (Chowdhury et al., 2018; Kitcher et al., 2019). For post-treatment ABRs, animals were anesthetized with 55 mg/kg ketamine and 5.5 mg/kg xylazine (females) or 70 mg/kg ketamine and 7.0 mg/kg xylazine (males), based on our observation that larger animals required higher doses to achieve sufficient anesthesia depth. Reversal agent was not administered. After ABR recordings, animals were euthanized by isoflurane inhalation (5%) followed by decapitation.

#### Rat cochlear dissections, labeling, and imaging

We removed rat temporal bones and fixed overnight in 4% PFA, rinsed the temporal bones three times in PBS, then decalcified in 120 mM ethylenediaminetetraacetic acid (pH 8) for up to 2 weeks prior to dissection and labeling. All solutions were diluted in PBS. First, we permeabilized cochleae in 0.5% NP40 for 10–60 min at 4°C, then rinsed in PBS and incubated in Alexa Fluor 488 phalloidin (1:100, catalog # F35355, Invitrogen) for 1 h. We again rinsed in PBS, then incubated in DAPI (1:1,000) for 10 min. After 2 PBS rinses, we mounted cochleae to bridged slides with Prolong Gold antifade reagent (ThermoFisher, Waltham, MA, United States). For imaging, we used a Leica SP8 confocal microscope with a 63× objective to collect images at the basal and middle turns. Hair cells were counted in 100 μM regions at these two locations.

### Statistical analysis

Data were analyzed by one- or two-way ANOVA, as appropriate, followed by Bonferroni-corrected *post hoc* tests for multiple comparisons. Analysis was performed in Prism v. 9. Data are presented as mean ± 1 s.d. unless otherwise stated.

## Results

### Some COVID-19 therapies are toxic to lateral line hair cells

**FIGURE 1:**
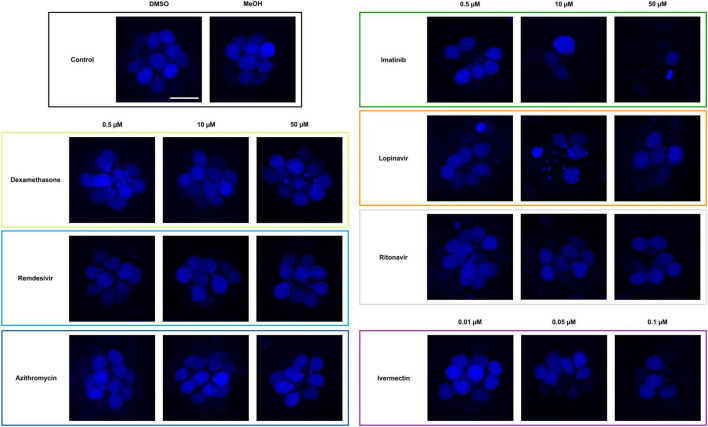
Some COVID-19 drugs are toxic to zebrafish hair cells. Hair cells were live-labeled with DAPI (blue), then fish were treated for 24 h in the indicated drug. The top left panel shows the vehicle controls with a full complement of hair cells. Methanol (MeOH) served as the vehicle for dexamethasone, while DMSO is the vehicle for all other drugs shown here. All other panels show representative confocal images for three concentrations of each drug. Left panels: non-ototoxic drugs dexamethasone, remdesivir, and azithromycin. Right panels: imatinib, lopinavir, ritonavir, and ivermectin all caused hair cell loss. Note that ivermectin was toxic to the animal at high concentrations, so only the non-lethal concentrations were used for this experiment. The scale bar in the upper left = 10 μm and applies to all panels.

**FIGURE 2:**
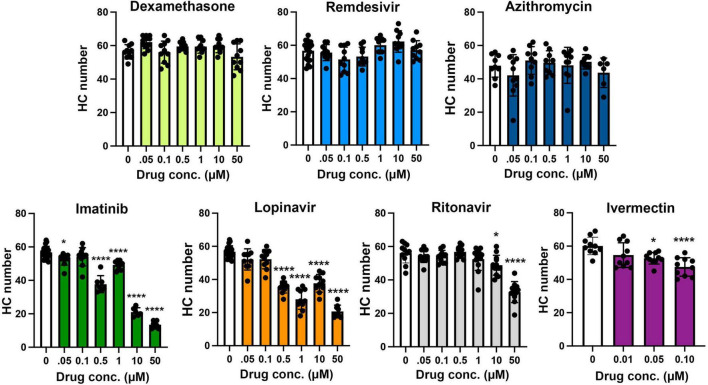
Hair cell quantification following treatment with COVID-19 therapies. Hair cells were counted from images represented in Figure 1. Controls (“0,” white bars) received vehicle, which was methanol for dexamethasone-treated fish and DMSO for all other drugs. Dexamethasone was used as a negative control. Hair cells were counted in five neuromasts per fish and summed to arrive at one value per animal. Data were analyzed by one-way ANOVA followed by Bonferroni-corrected *post hoc* tests. One-way ANOVA results and sample sizes are as follows: *Dexamethasone F*_(6_,_63)_ = 3.53, *p* = 0.0045, *N* = 10; *Remdesivir F*_(6_,_73)_ = 4.054, *p* = 0.0015, *N* = 10–20; *Azithromycin F*_(6_,_55)_ = 1.28, *p* = 0.281, *N* = 6–10; *Imatinib F*_(6_,_63)_ = 188.0, *p* < 0.0001, *N* = 6–20; *Lopinavir F*_(6_,_72)_ = 80.15, *p* < 0.0001, *N* = 9–20; *Ritonavir F*_(6_,_75)_ = 27.99, p < 0.0001, *N* = 11–13; *Ivermectin F*_(3_,_36)_ = 8.59, *p* = 0.0002, *N* = 10. Asterisks indicate significance differences from vehicle controls. *p < 0.05, ****p < 0.0001. Note that the imatinib and lopinavir experiments were run concurrently and therefore shared control animals. Data are represented as mean ± 1 s.d. and black dots represent individual fish.

We asked if seven drugs in clinical trials for COVID-19 had the potential to cause ototoxicity as a negative side-effect of drug administration. Drugs were selected based on clinical trials registered as of December 2020 when we commenced these experiments. Four of the seven drugs caused significant hair cell damage at one or more concentrations tested (Figures 1, 2). Neither remdesivir nor dexamethasone caused significant hair cell damage over the entire dose range examined (0.05–50 μM), nor did the antibiotic azithromycin (Figures 1, 2). By contrast, the tyrosine kinase inhibitor imatinib significantly damaged hair cells, with damage detected following 0.05 μM imatinib exposure (52.50 ± 3.37 HCs) as compared to vehicle controls (56.75 ± 3.64 HCs). Hair cell numbers decreased with increased drug concentration, with a 76% reduction in hair cell survival after treatment with 50 μM imatinib (13.50 ± 2.07 HCs) (Figure 2).

Both of the viral protease inhibitors lopinavir and ritonavir caused significant hair cell damage in the zebrafish lateral line. Of the two, lopinavir caused more severe damage at lower concentrations, with 38% hair cell loss observed following treatment with 0.5 μM lopinavir (35.40 ± 3.60 HCs). As with imatinib, hair cell death increased in a dose-dependent manner; only 36% of hair cells were present following administration of 50 μM lopinavir (20.67 ± 3.81 HCs). Ritonavir was not toxic at lower concentrations. Hair cell damage manifested first at 10 μM ritonavir (48 ± 5.94 HCs), with a maximum of 41% hair cell loss caused by 50 μM ritonavir (32.72 ± 6.28 HCs) (Figure 2).

Higher concentrations of ivermectin were toxic to larval zebrafish, with mortality beginning at 0.25 μM. Lower ivermectin concentrations did not cause overt morbidity as determined by observing swimming behavior and general morphology. However, 0.05 or 0.1 μM ivermectin were toxic to lateral line hair cells, with 12 and 21% hair cell loss, respectively (vehicle control: 60.10 ± 5.28 HCs; 0.05 μM: 52.70 ± 3.46 HCs; 0.1 μM: 47.50 ± 5.56 HCs) (Figure 2).

**FIGURE 3:**
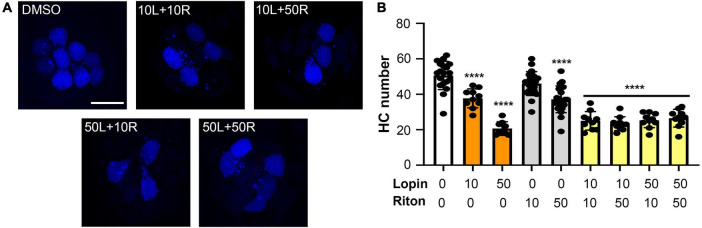
Hair cell toxicity of lopinavir and ritonavir combinations. DAPI-labeled fish were treated for 24 h with 10 and/or 50 μM of lopinavir (L), ritonavir (R), or both drugs. **(A)** Representative images of each drug combination; representative images of each drug alone are shown in Figure 1. Scale bar in the upper left panel = 10 μm and applies to all images. **(B)** There was a significant effect of drug treatment on hair cell number [one-way ANOVA, *F*_(8_,_112)_ = 42.68, *p* < 0.0001]. Asterisks indicate significant differences from vehicle controls (****p < 0.0001). There were no differences between fish treated with different combinations of both drugs (10/10, 10/50, 50/10, or 50/50). Statistical analysis for all pairwise comparisons is shown in Supplementary Table 1. Note that the 10 and 50 μM lopinavir data (without ritonavir) are also presented in Figure 2. Data are represented as mean ± 1 s.d. and black dots represent individual fish (*N* = 9–22/treatment).

Lopinavir and ritonavir are often administered in combination, both for COVID-19 clinical trials and as part of multi-drug cocktails in HIV patients. We therefore asked if the two drugs exhibited more hair cell damage in combination than when either drug was administered singly. As shown in Figure 3, 10 μM of both lopinavir and ritonavir combined caused 50% hair cell loss (vehicle control: 49.70 ± 10.04 HCs; 10 μM lopin/riton: 25.00 ± 5.23 HCs), significantly more damage than we observed with 10 μM lopinavir or ritonavir delivered alone (10 μM lopin: 37.70 ± 5.36 HCs, *p* < 0.001 compared to 10L/10R; 10 μM riton: 46.04 ± 6.73 HCs, *p* < 0.0001 compared to 10L/10R). However, there was no significant difference between 50 μM lopinavir and any of the lopinavir/ritonavir combinations. Please see Supplementary Table 1 for the pairwise analysis of all lopinavir/ritonavir combinations.

### Hair cell toxins can alter pre-synaptic ribbons

**FIGURE 4:**
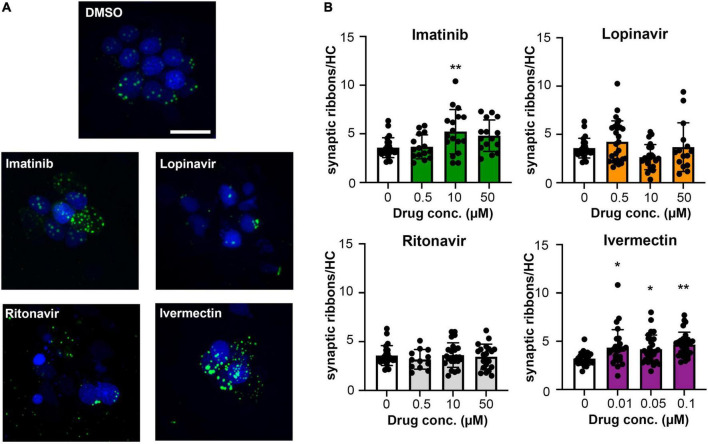
Some COVID-19 drugs alter the number of pre-synaptic ribbons. Rib-GFP fish were live-labeled with DAPI and treated with imatinib, lopinavir, ritonavir, or ivermectin for 24 h. **(A)** Representative confocal images of DMSO (vehicle) controls and the highest concentration of each COVID-19 drug (50 μM imatinib, lopinavir, and ritonavir; 0.1 μM ivermectin). Hair cell nuclei are labeled in blue, green punctae represent GFP + ribbons. The scale bar in the top image = 10 μm and applies to all panels. **(B)** Quantified GFP + punctae per remaining hair cell. Data were analyzed by one-way ANOVA followed by Bonferroni-corrected *post hoc* tests. One-way ANOVA results and sample sizes are as follows: *Imatinib F*_(3_,_66)_ = 4.916, *p* = 0.0038 (*N* = 5–6 fish, 15–17 neuromasts per group). *Lopinavir: F*_(3_,_83)_ = 3.140, *p* = 0.0296 (*N* = 7–10 fish, 15–30 neuromasts per group). *Ritonavir: F*_(3_,_75)_ = 0.4199, *p* = 0.7392 (*N* = 4–7 fish, 12–24 neuromasts per group). *Ivermectin: F*_(3_,_101)_ = 4.929, *p* = 0.0031 (*N* = 9 fish, 27 neuromasts per group). Asterisks indicate significance differences from vehicle (DMSO) controls. *p < 0.05, **p < 0.01. Note that the imatinib, lopinavir, and ritonavir experiments were run concurrently and therefore shared control animals. Control values for that experiment are not significantly different from control values from the ivermectin experiment (*t*-test, *p* = 0.15). Data are represented as mean ± 1 s.d. and black dots represent individual neuromasts.

In addition to hair cell death, drugs and other hearing toxins can cause sub-lethal damage to ribbon synapses, thereby reducing transmission from hair cells to afferent neurons (Kujawa and Liberman, 2009; Chen et al., 2012; Liu et al., 2013). We therefore asked if imatinib, lopinavir, ritonavir, or ivermectin reduced the number of pre-synaptic ribbons in surviving hair cells (Figure 4A). Vehicle-treated controls had an average of 3.5 ribbons/hair cell (Figure 4B), consistent with published reports in the lateral line (Obholzer et al., 2008; Nicolson, 2015). As seen in Figure 4, 24-h exposure to lopinavir or ritonavir did not impact the number of ribbons. Interestingly, there was a significant increase in the number of ribbons per hair cell in fish treated with either imatinib or ivermectin. Exposure to 10 μM imatinib increased the number of ribbons to 5.23 ± 1.61 per hair cell. Ivermectin exposure caused a dose-dependent increase in ribbon number that was significant at all concentrations tested, with 4.65 ± 1.29 ribbons/hair cell observed at the highest ivermectin dose (Figure 4B). These data suggest that some COVID-19 drugs impact synaptic transmission at the hair cell-afferent synapse.

### Specificity of cytotoxic effects

**FIGURE 5:**
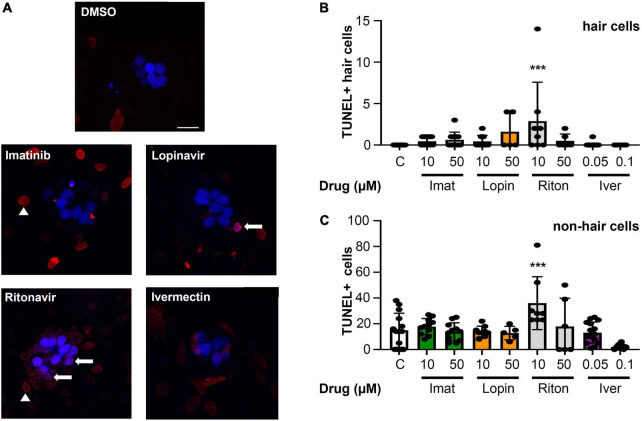
Most COVID-19 therapies do not cause overall cytotoxicity. **(A)** Representative images of TUNEL-labeled neuromasts. Hair cells are labeled with DAPI (blue), TUNEL + cells are red. Arrows show examples of DAPI + /TUNEL + hair cells, while arrow heads point to examples of DAPI-/TUNEL + cells (non-hair cells). Images show 10 μM imatinib, lopinavir, and ritonavir, and 0.05 μM ivermectin to show the range of labeling patterns we observed. The scale bar = 10 μm and applies to all panels. **(B)** Quantification of TUNEL + hair cells (double-labeled cells). There is a significant effect of treatment on the number of TUNEL + HCs [one-way ANOVA, *F*_(8_,_86)_ = 3.413, *p* = 0.0019]. **(C)** Quantification of TUNEL + non-hair cells (TUNEL + /DAPI) from the same images (62 μm × 62 μm box), to determine overall cytotoxicity of COVID-19 therapies. The x-axis in both panels **(B,C)** denotes the drug concentration (μM), and C = control. Note the difference in the y-axis scaling between **(B)** and **(C)** to reflect the higher number of TUNEL + non-hair cells as compared to hair cells. There is a significant treatment effect on the number of TUNEL + non-hair cells as well [one-way ANOVA, *F*_(8_,_78)_ = 5.061, *p* < 0.0001]. Asterisks indicate significant differences from DMSO controls ***p < 0.001. *N* = 5–23 fish/treatment. Bars represent mean ± 1 s.d.

While we observed significant hair cell loss from four of seven drugs tested, it’s possible that these drugs cause general cytotoxicity rather than specific damage to hair cells. We therefore used a TUNEL assay to quantify cell death in lateral line hair cells vs. surrounding non-sensory cells. As shown in Figure 5, there was no significant increase in overall cytotoxicity in imatinib, lopinavir, or ivermectin-treated fish. We also did not detect a significant increase in the number of TUNEL+ hair cells in these animals, suggesting hair cell death may have occurred prior to our 24-h sampling time point. 10 μM ritonavir significantly increased the number of both TUNEL+ hair cells and TUNEL+ non-hair cells, suggesting ongoing cell death in response to low-level ritonavir exposure.

### Mechanotransduction dependence of hair cell toxicity

**FIGURE 6:**
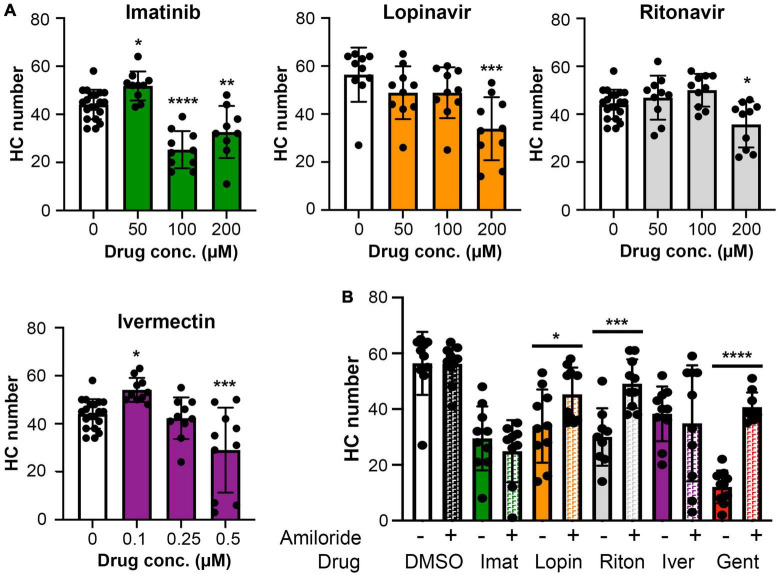
**(A)** High concentrations of imatinib, lopinavir, ritonavir, and ivermectin cause hair cell toxicity over a 6-h incubation period Data were analyzed by one-way ANOVA. Imatinib: *F*_(3_,_45)_ = 26.14, *p* < 0.0001. Lopinavir: *F*_(3_,_36)_ = 6.67, *p* = 0.0011. Ritonavir: *F*_(3_,_46)_ = 6.39, *p* = 0.001 Ivermectin: *F*_(3_,_46)_ = 11.14, *p* < 0.0001. *Post hoc* comparisons were conducted with Bonferroni-corrected *t*-test. Asterisks indicate significant differences from vehicle-only controls. *p < 0.05, **p < 0.01, ***p < 0.001, ****p < 0.0001. *N* = 9–20 fish per treatment. Note that the imatinib, ritonavir, and ivermectin experiments were run concurrently and therefore share vehicle (DMSO) controls. **(B)** MET channel block attenuates hair cell toxicity of some COVID-19 drugs. 1 mM amiloride was used to block MET channel function and gentamicin was used as a positive control. Paired *t*-tests (adjusted for multiple comparisons) were used to determine significant differences between amiloride (hashed bars) and non-amiloride (solid bars) treated fish within a single drug. DMSO *p* = 0.96; imatinib (imat) *p* = 0.39; lopinavir (lopin) *p* = 0.04; ritonavir (riton) *p* = 0.0004; ivermectin (iver) *p* = 0.64, gentamicin (gent) *p* < 0.0001. Asterisks indicate significant pairwise differences. *N* = 9–10 fish per treatment, bars represent mean ± 1 s.d.

Ototoxic aminoglycosides enter hair cells primarily via the MET channel and MET inhibition is sufficient to attenuate damage from some other hair cell toxins (Alharazneh et al., 2011; Thomas et al., 2013; Neveux et al., 2017). We therefore asked if MET channel function is necessary for hair cell damage caused by imatinib, lopinavir, ritonavir, or ivermectin. We inhibited MET function with the channel blocker amiloride, then co-administered amiloride and one of our four hair cell toxins. Preliminary experiments showed that 1 mM amiloride was toxic to hair cells over a 24-h period, while 6 h of amiloride exposure did not cause damage (Coffin et al., 2009 and data not shown). We therefore sought to determine if higher concentrations of COVID-19 drug were sufficient to cause hair cell damage after 6 h. Imatinib, lopinavir, or ritonavir at 200 μM was sufficient to cause hair cell loss, with a reduction of 26, 40, and 19% for imatinib, lopinavir, and ritonavir, respectively (Figure 6A). We observed hair cell loss with 100 μM imatinib as well, while lopinavir and ritonavir only damaged hair cells at the highest concentration tested. Significant hair cell damage was observed following 6-h ivermectin exposure, with 0.5 μM ivermectin causing 34% hair cell loss (Figure 6A).

We therefore used 200 μM imatinib, lopinavir, or ritonavir, or 0.5 μM ivermectin, for our MET inhibition experiments. 1 mM amiloride conferred significant protection from lopinavir or ritonavir damage, with complete protection observed in the amiloride + ritonavir-treated animals (Figure 6B). By contrast, amiloride provided no protection from imatinib or ivermectin toxicity (Figure 6B). As expected, amiloride significantly attenuated damage from the aminoglycoside gentamicin, and amiloride alone did not cause damage during the 6-h incubation period, consistent with our published data (Coffin et al., 2009). These data suggest that lopinavir and ritonavir may enter hair cells in a MET-dependent manner.

### Ivermectin was not ototoxic in rats

**FIGURE 7:**
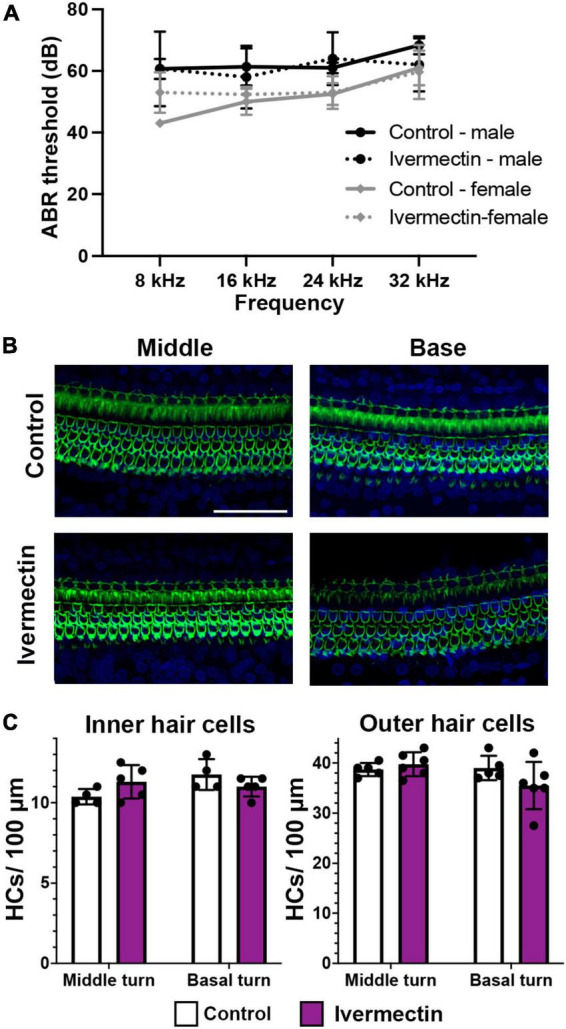
Ivermectin is not ototoxic to rats *in vivo*. **(A)** Post-treatment ABR thresholds for saline- and ivermectin-treated rats (control: solid line, ivermectin: dotted line; black lines and circles denote males, gray lines, and diamonds denote females). There is no significant treatment effect [two-way ANOVA, treatment effect, *F*_(1_,_36)_ = 0.046, *p* = 0.832]. **(B)** Representative confocal images from the middle and basal turns of control and ivermectin-treated rats. Phalloidin (green) labels hair bundles, DAPI (blue) labels nuclei. The scale bar in the upper right image = 50 μm and applies to all images. **(C)** Inner hair cell (left) and outer hair cell (right) quantification in the middle and basal cochlear turns. Counts were performed in both cochleae per animal and averaged. White bars represent control animals, purple bars ivermectin-treated animals. There was no significant difference in IHC number across treatments (middle turn: *p* = 0.073, basal turn: *p* = 0.097). Similarly, there was no significant difference in OHC number in either the middle or basal turns (paired *t*-tests, *p* = 0.202 and 0.084, respectively). Both male and female animals were included in the experiment and pooled for analysis. *N* = 5 control, *N* = 6 ivermectin. All quantitative data in panels **(A,C)** are presented as mean ± 1 s.d.

While the lateral line is an excellent model for early preclinical research, including ototoxicity, it is important to validate findings in a mammalian model. We therefore used rats to examine the ototoxicity of ivermectin. We selected ivermectin because it is the target of several clinical trials for COVID-19 and because it was sought off-label by individuals seeking an “alternative” COVID-19 therapy, causing the FDA and leading medical organization to label ivermectin a serious public health concern (Dyer, 2022; Meyerowitz-Katz et al., 2022). 12 days of 0.2 mg/kg ivermectin treatment did not cause significant hearing loss, as determined by ABR threshold values recorded at baseline, then recorded in the same animals following ivermectin treatment and a 2-week recovery period (Figure 7A). Consistent with these results, there was no loss of either inner or outer hair cells in the cochlea of ivermectin-treated rats (Figures 7B,C). Ivermectin-treated rats gained weight appropriately (Table 1) and behaved normally.

**TABLE 1:** There were no differences in weight gain between saline- and ivermectin-treated animals (2-tailed *t*-test, male *p* = 088, female *p* = 0.14).

		Start weight (g)	End weight (g)	% Weight gain
Saline	Male	122.67 ± 9.07	245.67 ± 15.88	100.36 ± 2.68
	Female	96.5 ± 0.71	152 ± 7.07	57.49 ± 6.17
Ivermectin	Male	127.67 ± 10.21	256.67 ± 17.56	101.37 ± 10.83
	Female	98.5 ± 3.11	150 ± 5.29	52.27 ± 1.33

Data are presented as mean ± 1 s.d.

## Discussion

Drugs such as aminoglycoside antibiotics and platinum-based chemotherapy agents cause permanent sensorineural hearing impairment, yet ototoxic potential is not commonly assessed during pre-clinical drug development or clinical trials. The COVID-19 pandemic has greatly increased the pace of drug discovery, including development of new drug candidates and repurposing existing drugs to combat this viral threat. With over 900 drugs in clinical trials for COVID-19 (Clinicaltrials.gov, 2022), it is highly likely that some pose a danger to the auditory system (Awad and Naimark, 2020; De Luca et al., 2021). We used the zebrafish lateral line as a model to assess the potential for seven COVID-19 drugs to cause hair cell damage.

Neither remdesivir nor dexamethasone, two drugs considered standard of care for severe COVID-19, damaged lateral line hair cells. Dexamethasone is considered “ear-safe” and is commonly used to treat sudden idiopathic sensorineural hearing loss (Egli Gallo et al., 2013; Hayward et al., 2019; Singh and Kumar Irugu, 2020; Hu et al., 2021), and we included this drug as a negative control. There are no case reports linking remdesivir to ototoxicity but related nucleotide analogs are associated with hearing loss (Jain et al., 2011). The macrolide antibiotic azithromycin also did not damage hair cells. Multiple studies report reversible hearing loss in patients taking azithromycin, while very few case reports of permanent hearing impairment exist [reviewed in Rybak et al. (2021)]. Collectively, these data suggest that azithromycin may transiently impact the auditory system but is not likely a hair cell toxin.

The retroviral drugs lopinavir and ritonavir both significantly damaged hair cells, as did the anti-cancer agent imatinib and the anti-parasitic drug ivermectin. Damage was apparent after 24 h at relatively low concentrations of each drug, while high concentrations caused significant hair cell loss within 6 h. We did not see hair cell damage after 1 h of treatment with 200 μM lopinavir or ritonavir (data not shown). These data suggest a cumulative dosing effect similar to cisplatin-induced hair cell toxicity (Li et al., 2004; Ou et al., 2007; Camet et al., 2021). By contrast, ototoxic agents such as neomycin cause rapid hair cell death (30–60 min in the lateral line), with minimal additional damage occurring over longer incubation periods (Coffin et al., 2009; Owens et al., 2009). These data suggest both similarities and differences between known ototoxins and putative damage mechanisms caused by COVID-19 drugs, as discussed below.

### Lopinavir-ritonavir

One case report in an HIV patient identified sensorineural hearing impairment following lopinavir-ritonavir therapy but there is little clinical data on the ototoxic potential of these drugs alone or in combination (Williams, 2001). Ritonavir reduced the viability of auditory cells *in vitro*, consistent with our data, but to our knowledge the ototoxic potential of lopinavir has not been examined (Thein et al., 2014). While either drug damaged hair cells, the two are generally administered together in clinical settings for both HIV patients and in COVID-19 trials. We therefore asked if combinatorial administration altered hair cell toxicity. Ritonavir (10 μM) caused modest damage (13%), while 10 μM lopinavir was a more potent hair cell toxin (33% HC loss). Combined administration reduced hair cell numbers by 50%, suggesting potential synergistic cytotoxicity at lower concentrations. However, there was no increase in damage when the two drugs were simultaneously administered at 50 μM, suggesting a possible ceiling effect.

Neither lopinavir nor ritonavir reduced the number of pre-synaptic ribbons, suggesting that these drugs do not cause synaptopathy. Lopinavir was not generally cytotoxic, despite causing substantial hair cell loss at relatively low concentrations; there was no increase in the number of TUNEL+ cells across the surface of the animal. By contrast, ritonavir significantly increased the number of apoptotic cells outside of the neuromast, suggesting some general cytotoxicity with ongoing exposure. While lopinavir and ritonavir are most often used in combination, studies in several cancer cell lines (e.g., urological cancers, acute myeloid leukemia) show that either lopinavir or ritonavir can be cytotoxic alone, depending on the cell line used (Johnson et al., 2011; Kraus et al., 2014; Okubo et al., 2019). Further studies are needed to determine identity of the TUNEL+ non-hair cells in rotinavir-treated fish and to understand the cytotoxic mechanisms.

Interestingly, MET channel inhibition with amiloride significantly protected hair cells from lopinavir or ritonavir treatment. The MET channel is the primary site for aminoglycoside entry into hair cells and channel inhibition prevents aminoglycoside-induced hair cell death (Marcotti et al., 2005; Coffin et al., 2009; Alharazneh et al., 2011). Our data suggest a similar entry mechanism for lopinavir and ritonavir, although it is possible that these drugs enter hair cells through an alternative MET-dependent route. While future experiments are needed to determine if these drugs interact with the MET channel, our data suggest that drug entry is required for hair cell damage.

While lopinavir-ritonavir treatment is a standard therapy in HIV patients, studies in the last decade have examined these drugs as a possible anti-cancer treatment (Dalva-Aydemir et al., 2015; Kast et al., 2016; Okubo et al., 2019; Marima et al., 2020). Lopinavir and ritonavir induce apoptosis in glioma, lung cancer, and other tumor cell lines, likely by downregulating pro-survival factors such as AKT while increasing endoplasmic reticulum (ER) and mitochondrial stress (Kast et al., 2016; Okubo et al., 2019; Marima et al., 2020). Lopinavir and ritonavir also demonstrate some cytotoxicity in in kidney proximal tubule cells, another target of aminoglycoside-induced damage (Walker et al., 1990; Humes, 1999; Vidal et al., 2006). Collectively, these studies suggest that lopinavir and ritonavir may induce ototoxicity *via* similar mechanisms as aminoglycosides, including MET-dependent entry and induction of ER and mitochondrial stress leading to hair cell apoptosis (Alharazneh et al., 2011; Böttger and Schacht, 2013; Esterberg et al., 2013, 2014).

It’s unknown if lopinavir and/or ritonavir pose an ototoxic risk in mammals. Lopinavir was detected in postmortem brain tissue from HIV patients and ritonavir is associated with microglial activation in this same patient population (Soontornniyomkij et al., 2018; Ferrara et al., 2020), suggesting the drug can cross the blood-brain barrier (BBB) and therefore may also cross the blood-labyrinth barrier (BLB) and gain access to the inner ear. Based on clinical trial results, lopinavir-ritonavir is not effective for COVID-19, although some clinical trials were still recruiting as of April 2022 (Ader et al., 2021; Reis et al., 2021; Clinicaltrials.gov, 2022; Hafez et al., 2022). However, given the common use of this drug combination in HIV patients and the potential for cancer therapy, it’s important to further understand the ototoxic potential of lopinavir-ritonavir treatment in a mammalian model.

### Imatinib

Imatinib caused significant hair cell damage across a wide range of concentrations, with substantial damage observed beginning at 0.5 μM (24-h exposure). Imatinib is a first-line therapy for chronic myeloid leukemia (CML), a form of cancer caused by chromosomal fusion of BCR and ABL1 (Ren, 2005). Fusion results in constitutive activation of an oncogenic tyrosine kinase, facilitating tumor growth. Imatinib specifically inhibits the non-receptor tyrosine kinase ABL1 and reduces kinase activity (Ren, 2005). Imatinib-induced hair cell death was previously noted in a zebrafish screen of chemotherapeutic agents (Hirose et al., 2011) and multiple case reports point to this drug as a likely ototoxin (Attili et al., 2008; Lin et al., 2012; Wasif et al., 2016). However, the mechanisms of hair cell damage are unknown.

Wildtype Abl1 is widely expressed and plays a role regulating cell proliferation and survival, including regulating cell death pathways in response to cellular stressors (Ren, 2005; Greuber et al., 2013; Wang, 2014). It’s therefore possible that imatinib damages hair cells by directly interacting with Abl1. However, imatinib treatment in cancer cells reduces expression of pro-survival factors including Bcl-X_*L*_ and Bcl-2 (Brauer et al., 2007); Bcl-2 family proteins play a role in hair cell survival from known ototoxins such as aminoglycosides (Cunningham et al., 2004; Coffin et al., 2013). Therefore, imatinib may also act indirectly by altering the balance of pro-survival and pro-apoptotic factors. Damage was independent of MET channel activity, suggesting that either the drug enters hair cells via an alternate route or that damage is initiated extracellularly, perhaps by imatinib binding to a membrane-bound receptor tyrosine kinase.

Interestingly, imatinib treatment significantly increased the number of pre-synaptic ribbons in surviving hair cells. While the mechanism underlying this phenomenon is unknown, it may be due to dysregulation of hair cell calcium levels. Imatinib disrupts calcium homeostasis in blood cells taken from CML patients and in cardiomyocytes, in part by acting on mitochondria, a major intracellular calcium store (Ciarcia et al., 2012; Barr et al., 2014; Varga et al., 2015). Intracellular calcium regulates synaptic ribbon size and number in hair cells during development and following acoustic over-exposure (Sheets et al., 2012, 2017; Sebe et al., 2017; Wong et al., 2019; Liu et al., 2021). However, disruption of calcium homeostasis generally leads to a reduction in the number of pre-synaptic ribbons, rather than the increase observed here. It is also possible that imatinib leads to ribbon fragmentation, which would manifest as an increase in ribbon punctae using our quantification method. More research is needed to determine if imatinib alters calcium regulation in hair cells, with subsequent impacts on pre-synaptic ribbons and hair cell survival.

### Ivermectin

Ivermectin is commonly used for parasitic infections, including ear mites and heartworm prevention in dogs, as a dewormer for horses, and treatment of infections such as scabies and river blindness in humans (Laing et al., 2017). A 2020 paper demonstrated that ivermectin reduced SARS-CoV-2 replication in cell culture (Caly et al., 2020), leading to a series of clinical trials for ivermectin as both a therapy for COVID-19 patients and as a potential prophylactic to prevent SARS-CoV-2 infection (López-Medina et al., 2021; Vallejos et al., 2020). These clinical trials report little to no therapeutic efficacy for ivermectin in COVID-19 disease and high-profile organizations such as the US Food and Drug Administration and the National Institutes of Health have published warnings against off-label use of this drug (Cruciani et al., 2021; López-Medina et al., 2021; Lim et al., 2022; Molnar et al., 2022). However, ivermectin has gained notoriety as an “alternative” COVID-19 therapy, accentuating the need to understand the potential side-effects of ivermectin exposure.

High concentrations of ivermectin were generally toxic to the fish, while concentrations below 250 nM caused hair cell death without overt morbidity; larvae swam normally and there was no significant increase in the overall number of apoptotic cells. In vertebrates, ivermectin primarily targets GABA receptors but can also act on glycinergic and purinergic receptors (Soderlund et al., 1987; Sánchez-Nogueiro et al., 2009; Plested, 2016). By agonizing these targets, ivermectin generally acts to increase neural inhibition, consistent with literature reporting ivermectin neurotoxicity in rodents and other animals (Trailovic and Nedeljkovic, 2011; Salam et al., 2022).

Similar to imatinib, ivermectin treatment significantly increased the number of pre-synaptic ribbons in surviving hair cells. Synaptopathy generally results from increased glutamatergic transmission, leading to excitotoxic responses and a reduction in pre-synaptic ribbons and post-synaptic glutamate receptors (Liberman and Kujawa, 2017; Sebe et al., 2017; Kim et al., 2019). An increase in ribbon number is unusual and could reflect increased synaptic inhibition via GABAergic signaling. GABA receptors are expressed in zebrafish lateral line hair cells and in multiple cell types within the mammalian cochlea, including outer hair cells and type I afferent neurons (Plinkert et al., 1989; Arnold et al., 1998; Wedemeyer et al., 2013; Toro, 2018; Manchanda et al., 2021). GABA agonism could reduce glutamatergic signaling, initiating a compensatory mechanism that results in more excitatory synapses. Future experiments will examine the mechanisms of ivermectin-induced synaptic damage.

In contrast to the data in zebrafish, ivermectin did not cause hearing damage *in vivo* in rats. There are several possible explanations for this apparent discrepancy. Ivermectin may not cross the BLB and therefore not gain access to the cochlear compartment. Ivermectin crosses the BBB in multiple vertebrate groups, suggesting it may also cross the BLB (Katharios et al., 2004; Li et al., 2014; Chandler, 2018). Alternatively, this discrepancy between zebrafish and rodent results may have occurred because ototoxicity in rats was assessed *via* ABR recordings, which are not sufficient to detect synaptopathy (Kujawa and Liberman, 2009). Synaptic damage is also known to precede hair cell death, so ivermectin could impact ribbon synapses before ABR threshold shift or hair cell damage is observed (Wu et al., 2019; Fernandez et al., 2020).

Case reports have linked ivermectin to vestibular dysfunction, particularly dizziness (Bussaratid et al., 2005; Chandler, 2018). Therefore, it’s possible that ivermectin caused damage to vestibular epithelia. We did not see any behavioral changes associated with vestibular dysfunction; animals righted themselves normally, did not circle, were active in their home cages, and gained weight. Our dose of 0.2 mg/kg ivermectin was conservative and reflected doses used in clinical trials early in the pandemic (Vallejos et al., 2020; Cruciani et al., 2021; and NCT04391127, NCT04646109, NCT04591600). More recent trials have used ivermectin doses 2-6 times higher (e.g., Buonfrate et al., 2022; Lim et al., 2022). Despite the lack of robust clinical efficacy data, ivermectin trials in COVID-19 patients are still actively recruiting (28 studies as of April 2022, clinicaltrials.gov). Given our zebrafish data, future studies should examine higher, clinically relevant doses of ivermectin for both cochlear and vestibular toxicity using more sensitive measures.

## Conclusion

Our data demonstrate that lopinavir, ritonavir, imatinib, and ivermectin cause significant hair cell damage in zebrafish. All four drugs were considered as potential therapies for COVID-19, and all four are currently FDA-approved for other indications, including HIV (lopinavir-ritonavir), CML (imatinib), and parasitic infections (ivermectin). Therefore, ototoxic monitoring may be warranted for patients receiving these medications. Future studies are necessary to determine the mechanisms of hair cell toxicity and the degree to which each drug impacts the mammalian auditory periphery.

## Data availability statement

The raw data supporting the conclusions of this article will be made available by the authors, without undue reservation.

## Ethics statement

The animal study was reviewed and approved by Washington State University Animal Care and Use Committee.

## Author contributions

AC proposed the experiments and performed the bulk of the data analysis. AC, FF, JH, TH, and OM designed the experiments. All authors conducted the experiments, wrote and edited portions of the manuscript, and approved the submitted version.
