# Supplementary material for: Putative COVID-19 therapies imatinib, lopinavir, ritonavir, and ivermectin cause hair cell damage: A targeted screen in the zebrafish lateral line
Source: Front Cell Neurosci. 2022 Aug 24;16:941031. doi: 10.3389/fncel.2022.941031 (PMC9448854; doi:10.3389/fncel.2022.941031)
Supplement: Supplementary file 1 [file Data_Sheet_1.docx]

Coffin et al. Supplemental Figures


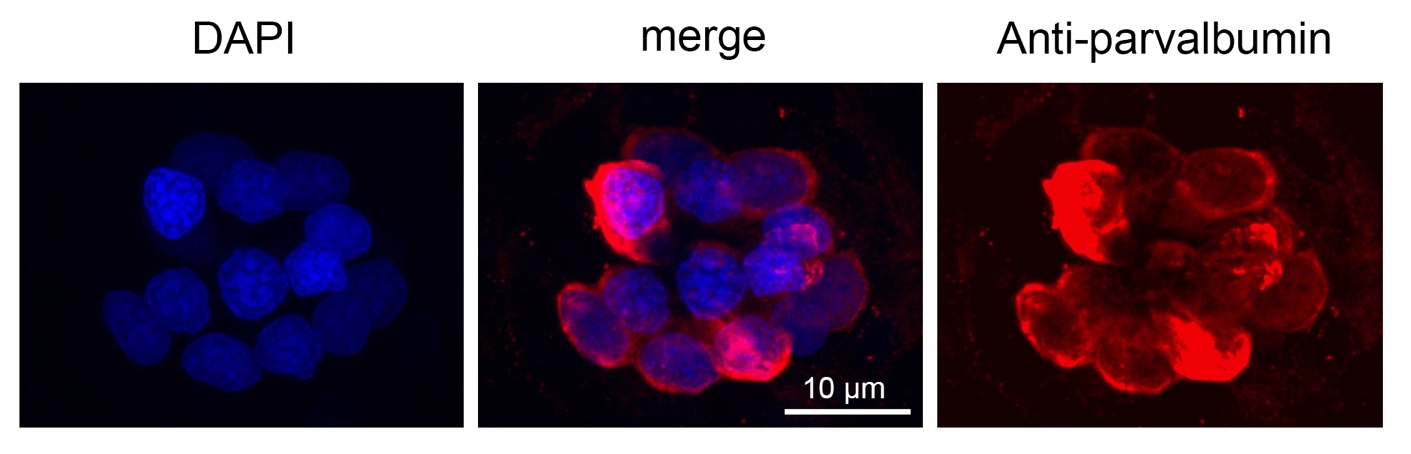


**Suppl Fig 1.** Corroboration of DAPI labeling with a second validated hair cell marker. Live larval zebrafish were labeled with DAPI as described in the Methods section, then euthanized with MS-222 and fixed in 4% paraformaldehyde. Larvae were then labeled with a mouse antibody to parvalbumin (1:500 dilution, MAB1572, Millipore Sigma). Parvalbumin is robustly expressed in lateral line hair cells and this antibody is commonly used as a hair cell marker (*e.g.,* McDermott et al. 2011; Coffin et al. 2013; Hayward et al. 2019; Shahab et al. 2021). Labeling was visualized with goat anti-mouse Alex 568 secondary antibody (1:500 dilution, A11011, Life Technologies). The full labeling protocol is available in Hayward et al. 2019. The left-hand image shows DAPI labeling, the right hand-image shows the same neuromast labeled with anti-parvalbumin, and the central image represents the merge of both labels. Images show neuromast M2; similar images were generated with other neuromasts.
