# Supplementary material for: Putative COVID-19 therapies imatinib, lopinavir, ritonavir, and ivermectin cause hair cell damage: A targeted screen in the zebrafish lateral line
Source: Front Cell Neurosci. 2022 Aug 24;16:941031. doi: 10.3389/fncel.2022.941031 (PMC9448854; doi:10.3389/fncel.2022.941031)
Supplement: Supplementary file 2 [file Table_1.docx]

|  | 0 | 10L | 50L | 10R | 50R | 10L/10R | 10L/50R | 50L/10R | 50L/50R |
| --- | --- | --- | --- | --- | --- | --- | --- | --- | --- |
| 0 |  | **** | **** | ns | **** | **** | **** | **** | **** |
| 10L |  |  | **** | * | ns | *** | **** | *** | ** |
| 50L |  |  |  | **** | **** | ns | ns | ns | ns |
| 10R |  |  |  |  | *** | **** | **** | **** | **** |
| 50R |  |  |  |  |  | **** | **** | **** | *** |
| 10L/10R |  |  |  |  |  |  | ns | ns | ns |
| 10L/50R |  |  |  |  |  |  |  | ns | ns |
| 50L/10R |  |  |  |  |  |  |  |  | ns |
| 50L/50R |  |  |  |  |  |  |  |  |  |

**Supplemental Table 1.** Statistical analysis for the lopinavir-ritonavir combinatorial experiment shown in Fig. 3. As stated in the legend for Fig. 3, these drugs caused significant hair cell loss (one-way ANOVA, F _8,112_ =42.68, p < 0.0001). Significant pairwise comparisons are shown here for all drug combinations (Bonferroni-corrected t-tests). ns = not significant, *p < 0.05, **p < 0.01, ***p < 0.001, ****p < 0.0001. L=lopinavir, R=ritonavir, numbers in the top and left-most columns indicate the concentration of each drug (e.g., 10L = 10 µM lopinavir).
